# Detailed morphological characterisation of Hendra virus infection of different cell types using super-resolution and conventional imaging

**DOI:** 10.1186/s12985-014-0200-5

**Published:** 2014-11-27

**Authors:** Paul Monaghan, Diane Green, Jackie Pallister, Reuben Klein, John White, Catherine Williams, Paul McMillan, Leann Tilley, Marko Lampe, Pippa Hawes, Lin-Fa Wang

**Affiliations:** CSIRO Australian Animal Health Laboratory, 5 Portarlington Road, Geelong, VIC 3220 Australia; Department of Biochemistry and Molecular Biology, Melbourne, Australia; ARC Centre of Excellence for Coherent X-ray Science, Melbourne, Australia; Bio21 Molecular Science and Biotechnology Institute, University of Melbourne, Melbourne, VIC 3010 Australia; Current Address: Biological Optical Microscopy Platform, Bio21 Molecular Science and Biotechnology Institute, University of Melbourne, Melbourne, VIC 3010 Australia; Leica Microsystems, CMS GmbH, Ernst-Leitz Strasse 17-37, Wetzlar, Germany; Current Address: European Molecular Biology Laboratory, Meyerhofstr 1, D-69117 Heidelberg, Germany; Current Address: Translational Lung Research Center (TLRC), Department Translational Pulmonology, University of Heidelberg, Im Neuenheimer Feld 350, D-69120 Heidelberg, Germany; Pirbright Institute, Pirbright, Woking, Surrey GU240NF UK; Duke-NUS Graduate Medical School, Singapore, Singapore

**Keywords:** Hendra virus, Paramyxovirus, Confocal microscopy, Super-resolution microscopy, M protein, G protein, Cell lines

## Abstract

**Background:**

Hendra virus (HeV) is a pleomorphic virus belonging to the *Paramyxovirus* family. Our long-term aim is to understand the process of assembly of HeV virions. As a first step, we sought to determine the most appropriate cell culture system with which to study this process, and then to use this model to define the morphology of the virus and identify the site of assembly by imaging key virus encoded proteins in infected cells.

**Methods:**

A range of primary cells and immortalised cell lines were infected with HeV, fixed at various time points post-infection, labelled for HeV proteins and imaged by confocal, super-resolution and transmission electron microscopy.

**Results:**

Significant differences were noted in viral protein distribution depending on the infected cell type. At 8 hpi HeV G protein was detected in the endoplasmic reticulum and M protein was seen predominantly in the nucleus in all cells tested. At 18 hpi, HeV-infected Vero cells showed M and G proteins throughout the cell and in transmission electron microscope (TEM) sections, in pleomorphic virus-like structures. In HeV infected MDBK, A549 and HeLa cells, HeV M protein was seen predominantly in the nucleus with G protein at the membrane. In HeV-infected primary bovine and porcine aortic endothelial cells and two bat-derived cell lines, HeV M protein was not seen at such high levels in the nucleus at any time point tested (8,12, 18, 24, 48 hpi) but was observed predominantly at the cell surface in a punctate pattern co-localised with G protein. These HeV M and G positive structures were confirmed as round HeV virions by TEM and super-resolution (SR) microscopy. SR imaging demonstrated for the first time sub-virion imaging of paramyxovirus proteins and the respective localisation of HeV G, M and N proteins within virions.

**Conclusion:**

These findings provide novel insights into the structure of HeV and show that for HeV imaging studies the choice of tissue culture cells may affect the experimental results. The results also indicate that HeV should be considered a predominantly round virus with a mean diameter of approximately 280 nm by TEM and 310 nm by SR imaging.

## Background

Hendra virus (HeV), along with the closely-related Nipah virus (NiV) and Cedar virus (CedPV), form the *Henipavirus* genus in the family *Paramyxoviridae*. Bats are the reservoir host for the henipaviruses and have been the source of a number of spill-over events. HeV outbreaks have so far been restricted to northern Australia [1], but NiV outbreaks have occurred in Bangladesh, Malaysia/Singapore and India [2]. In these spill-over events domestic animals and humans are infected with significant mortality rates which, for NiV in particular, range from 40-100% [3].

Paramyxoviruses replicate within the host cell cytoplasm and virus particles bud from the cell surface, incorporating a portion of the host cell membrane as the viral envelope. However, the precise mechanisms involved in viral protein intra-cellular trafficking and infectious particle assembly are not clear for many viruses [4], including paramyxoviruses [5].

The HeV genome codes for 6 major proteins. The nucleoprotein (N), phosphoprotein (P) and polymerase (L) proteins interact with the newly formed RNA genome to form a ribonucleoprotein complex (RNP). In addition to the P protein, the P gene also encodes several smaller proteins [6]. Sub-cellular localisation of these V, W and C proteins has been demonstrated in infected cells with C and V proteins present throughout the cytoplasm and W protein in the nucleus (but not the nucleolus). All three proteins were also detected in purified virions [7]. HeV V, W and C proteins are present in relatively low abundance and their functions remain unclear, although they have been shown to inhibit transcription and replication [8].

Most work on henipavirus proteins in infected cells has focussed on the F and G glycoproteins found on the outside of the virions as they are key to the attachment and internalisation processes of the virus. The HeV G glycoprotein binds to its cell surface receptors ephrin B2 and ephrin B3 [9-11] which are most highly expressed on neurons, arterial endothelial and smooth muscle cells [12-14]. The F (fusion) glycoprotein undergoes a conformational change when G binds to a host cell and drives the fusion of the virion with the host cell membrane [15] to initiate the process of virus replication. The F proteins of both NiV and HeV have been shown to be synthesised in an inactive form and need activation by cathepsins which may take place within the endosomal compartment [16,17].

The HeV matrix protein (M), by analogy with other paramyxoviruses, is crucial for virion morphogenesis and along with the RNP constitutes the virion contents. The precise role that M protein plays in viral morphogenesis is unclear, although expression of NiV M protein in tissue culture cells leads to the formation of virus-like particles [18] and in *E. coli* the formation of round particles sized between 20 and 50 nm [19]. Patch et al. [20] identified a short sequence of NiV M protein that was critical for budding of viral–like particles. NiV M protein, along with the M protein of a small number of other paramyxoviruses [21-24] is found within the nucleus of infected cells, but the precise reason(s) for this are not clear. In their studies, [25] Wang et al. observed NiV M protein first in the nucleus and then later in infection, within the cytoplasm and at the plasma membrane. Furthermore, this transit through the nucleus appeared to be essential for correct viral budding. These authors also demonstrated that ubiquitination of NiV M protein takes place within the nucleus, and that this appears to be important for virus budding. In cells infected with respiratory syncytial virus (RSV), there was a reduction in host cell transcription raising the possibility that this may be a function of nuclear localised M protein [21].

An understanding of virion structure is a key stage in the process of unravelling henipavirus assembly. We used confocal and transmission electron microscopy (TEM) to compare HeV protein and virion production in different cell lines. In addition, two systems of super-resolution (SR) imaging were used to determine if sub-virion resolution of paramyxovirus proteins was feasible. These observations led to important conclusions regarding the morphology of HeV virions and the suitability of various cell lines as *in vitro* models of HeV replication.

## Results

### HeV M and G protein in HeV-infected Vero cells

**Figure 1:**
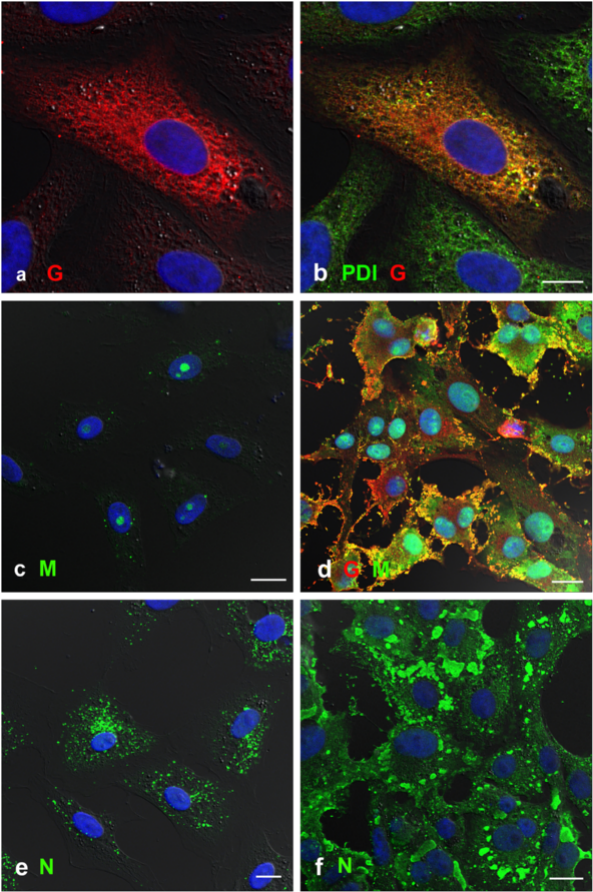
**Confocal images of Vero cells infected with HeV and fixed at 8 hpi (a, b, c, e) and 18 hpi (d, f. (a, b)** Cells were labelled with anti-HeV G mAb m102.4 [41] and mouse anti-PDI detected with species-specific immunoglobulins conjugated to Alexa 568 (HeV G red: **a, b**) and Alexa 488 (PDI; green, **b**, **c)** Cells were labelled with anti-HeV M antibody detected with anti-mouse antibody conjugated to Alexa 488 (green) showing M protein mainly in the nucleolus. **d)** Vero cells infected with HeV and fixed at 18 hpi. HeV M protein (green) was present in the nucleus but also throughout the cytoplasm and at the cell membrane. HeV G protein (labelled as in a, b: red) was present throughout the cell cytoplasm. **e)** HeV N protein (Alexa 488: green) was present at 8 hpi in punctate regions throughout the cytoplasm and in large accumulations at 18 hpi **(f)**. Nuclei were labelled with DAPI (blue). Fluorescence images were merged with DIC images. Scale bars: **a, b)** =10 μm, **c)** =15 μm, **d)** =20 μm, **e)** =10 μm, **f)** =20 μm.

**Figure 2:**
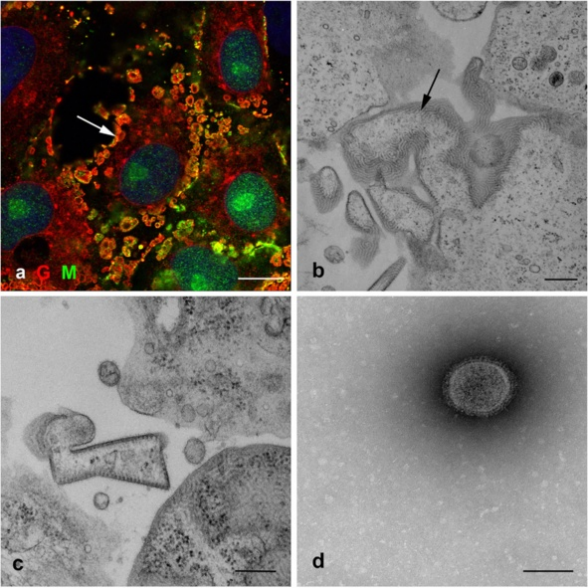
**Vero cells infected with HeV and fixed at 18 hpi for confocal and electron microscopy. a)** Confocal image of cells labelled with anti-HeV G mAb m102.4 and mouse anti HeV M antibodies detected with species-specific immunoglobulins conjugated to Alexa 488 (M, green) and 568 (G, red) showing regions of the cell where there is co-localisation of the two proteins (yellow, arrow). Nuclei were labelled with DAPI (blue) **b, c)** HeV-infected Vero cells processed for TEM showing RNPs accumulating at the cell membrane (**b**, arrow) and highly pleomorphic structures **(c, d)** a negative contrast TEM image of a preparation from an HeV-infected Vero culture. Scale bars: **a)** =10 μm, b =300 nm, c =300 nm, d =200 nm.

We postulated that co-localisation of the two HeV proteins M and G as shown by confocal microscopy would indicate either the site of virus assembly or the presence of individual viral particles in infected cell cultures. Vero cells were infected at an MOI of 8 then fixed at 8, 18 and 24 hours post infection (hpi) and labelled with antibodies to HeV N, M and G. At 8 hpi, HeV G protein was located within the cytoplasm in an endoplasmic reticulum (ER)-like pattern. Co-labelling with antibodies against an enzyme found in the ER, protein disulphide isomerase (PDI), showed almost complete co-localisation with the G protein confirming G protein synthesis within the ER (Figure 1a, b). In contrast, HeV M was localised within infected cell nuclei, mostly within the nucleoli (Figure 1c). The HeV M and G proteins were not co-localised at this time. By 18 hpi there were large numbers of syncytia throughout the culture with extensive expression of both M and G proteins throughout the cell cytoplasm and at the cell membrane (Figure 1d). HeV N protein was distributed throughout the cytoplasm in small punctate spots at 8 hpi (Figure 1e) and by 18 hpi, was present in large amounts throughout the cell cytoplasm and in small ‘lakes’ (Figure 1f). Figure 2a shows a typical area of infection at higher magnification illustrating HeV M and G proteins (at 18 hpi) apparently associated with bleb-like structures on the surface of the cell. It is not clear which of the structures in the image are virions. Figures 2b and 2c show a similar region as shown by TEM. Membrane-bound structures of variable shapes and sizes contain a large number of small tube-like ribonucleoprotein complexes (RNPs) (Figure 2b arrow). It is not clear if these structures are HeV infectious particles or membrane extrusions of the infected cells.

Although there was no obvious identification of infectious particles in HeV-infected Vero cells they are routinely used to produce infectious virus stocks. The morphology of virus-like particles present in these preparations was investigated using negative contrast electron microscopy. Most of the intact structures observed using this technique were round with a mean diameter of approximately 242 nm (n =15, SD 43) (Figure 2d).

### HeV M and G protein in A549, MDBK and HeLa cells

**Figure 3:**
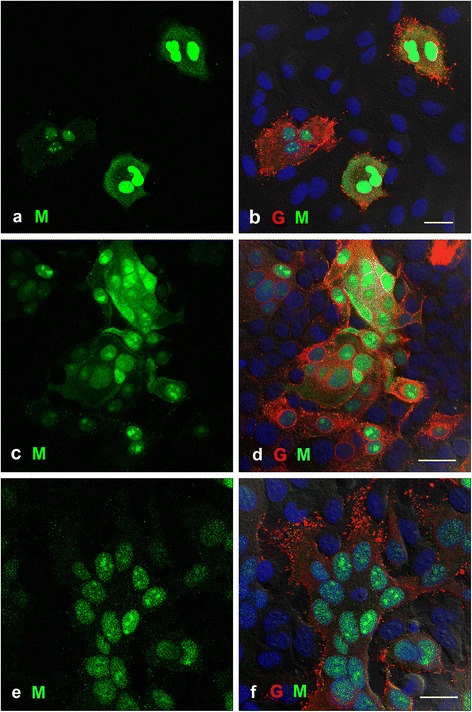
**Cells infected with HeV at an MOI of 8, fixed at 18hpi and labelled for confocal microscopy as in Figure** **1** **.** HeV M protein is shown in green and HeV G protein is shown in red. Cell nuclei are blue. **a, b)** A549 cells showing the majority of the HeV M protein in the nuclei **(a)**, with HeV G protein mainly at the cell membrane **(b)**. **c,d)** MDBK cells showing HeV M protein in the nuclei and cytoplasm **(c)**, with the HeV G protein predominantly at the cell membrane **(d)**. **e, f)** HeLa cells showing low levels of expression of HeV M protein in the nuclei with very little at the cell membrane **(e)**. HeV G protein is present predominantly at the cell surface **(f)**. The centre of the image shows multiple nuclei within a syncytium. Scale bars =20 μm.

To determine if the patterns of HeV M and G protein seen in Vero cells were typical of HeV-infected cells in general, A549, MDBK and HeLa cell lines were infected and fixed at 8 and 18 hpi. Whilst there were small differences between these cell lines, in HeV-infected cells fixed at 18 hpi the overall pattern observed consisted of HeV G protein at the cell membrane and the majority of the HeV M protein in the nucleus with some diffusely located in the cytoplasm. In A549 cells, the majority of G protein was not co-localised with M protein, although there were small numbers of co-localised dots at the cell membrane which probably represent virions (Figure 3a, b). In MDBK cells the HeV M protein was seen at high levels in both the nucleus and the cytoplasm (Figure 3c, d) but again very little M protein was observed at the cell membrane. HeV G protein was present at the cell membrane but only a small proportion appeared to be in association with M protein. In HeLa cells, as shown in Figure 3e, f, whilst the infection had progressed significantly to form a large syncytium containing around 15 nuclei in the centre of the image, the level of HeV M protein expression was low, with the majority located within the nucleus. Small amounts of M protein were seen at the cell membrane in a punctuate pattern. The majority of the HeV G protein was located at the cell membrane, with very little present within the cytoplasm.

### HeV M and G protein in bovine and porcine aortic endothelial cells

**Figure 4:**
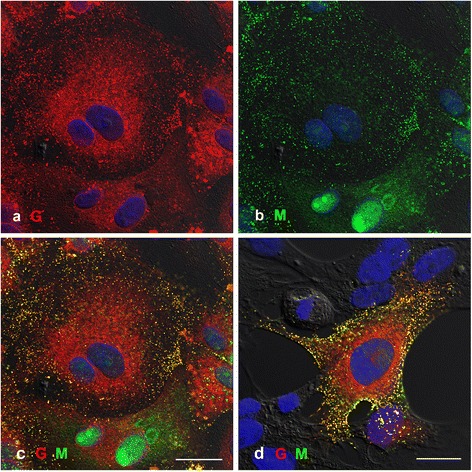
**Porcine aortic endothelial cells (PAEC) and bat-derived cells (PaLu) infected with HeV and fixed at 18 hpi for confocal microscopy. a)** PAEC cells labelled with anti-HeV G antibody mAb m102.4 detected with Alexa 568-conjugated anti-human antibody (red) showing an ER-like distribution of the HeV G protein and small dots. **b)** PAEC cells labelled with mouse anti-HeV M protein detected with anti-mouse antibody conjugated to Alexa 488 (green) **c)** overlaid image of **a)** and **b)** showing colocalisation of the M and G in the dots (yellow). **d)** PaLu cells labelled as for PAEC cells. Image is merged M (green) and G (red). Nuclei were labelled with DAPI (blue). Fluorescence images were merged with DIC images. Scale bars: **a, b, c)** =20 μm **d)** =20 μm.

As one of the major targets for henipaviruses *in vivo* are endothelial cells [26], primary cultures of both porcine (PAEC) and bovine (BAEC) aortic endothelial cells were prepared and infected with HeV. These species were initially selected on the basis of their published susceptibility to both HeV and NiV [27]. They were fixed at 8, 18, 24 and 48 hpi and labelled with individual antibodies specific for HeV M and G proteins. There were no significant differences seen in the expression of HeV M and G proteins between porcine and bovine endothelial cells. In both cell types, at 8 hpi, HeV G was seen in an ER-like pattern and low levels of M labelling were seen in a few nuclei (data not shown). However, at 18 hpi there was a significant difference in M and G expression in PAEC and BAEC cells when compared with Vero cells (Figures 1 and 4). In the endothelial cells, HeV G protein was present in an ER-like pattern but also as dots on the cell surface (Figure 4a). The HeV M protein was predominantly seen in dots on the cell surface and at low levels in some of the nuclei (Figure 4b). The individual labelling patterns for HeV M and G co-localised in the dots on the cell surface (Figure 4c). Interestingly, in cells where there were significant numbers of dots on the cell surface there appeared to be a lower level of M labelling in the nucleus. Importantly, at no time point tested was the pattern of HeV M and G proteins seen in the immortalised cell lines (see above) observed in HeV-infected primary endothelial cells.

### HeV M and G protein in bat kidney and bat lung-derived cell lines

Bats are the reservoir hosts of HeV and NiV, yet appear to be resistant to virus-induced disease. Two bat cell lines derived from bat kidney and bat lung (PaKi, PaLu) [28] were selected. They were infected with HeV, fixed at 8, 18 and 24 hpi and antibody labelled to detect HeV M and G proteins. Hendra virus M protein was present in the nuclei at low levels at 8 hpi and then predominantly at the cell membrane in small dots at 18 hpi. Hendra virus G protein was difficult to detect at 8 hpi but was present in an ER-like pattern and in dots at the cell membrane at 18 hpi. The M and G protein positive labelled dots at the surface of the cell showed clear co-localisation of the two proteins (yellow, Figure 4d), as seen in the endothelial cell cultures.

### Sub-virion resolution imaging of HeV N M and G proteins

The limit of resolution in light microscopy is around 250 – 300 nm in the xy plane, and ~500 nm in the z plane, making it difficult to define the dots seen in the confocal microscope as viral particles. Recent developments in SR imaging have significantly improved the resolution achievable using light microscopy. Based upon the data obtained, we hypothesised that primary endothelial cells would be a better model system than immortalised cell lines based upon their limited tissue culture history and noted that the HeV protein expression pattern was markedly similar in both species of endothelial cells as well as the bat-derived kidney cells. We chose these cells, therefore, for further SR experiments. Coverslips of PAEC and PaKi cells were infected with HeV, fixed at 18 hpi and prepared for confocal and two different systems of SR microscopy. Coverslips were labelled with individual antibodies recognising HeV G and N proteins which were detected with species-specific immunoglobulins as for confocal imaging. These proteins were selected as their distribution, as judged from other paramyxoviruses, should be within the viral membrane envelope (HeV N) and forming the glycoprotein coat on the virion exterior (HeV G).

**Figure 5:**
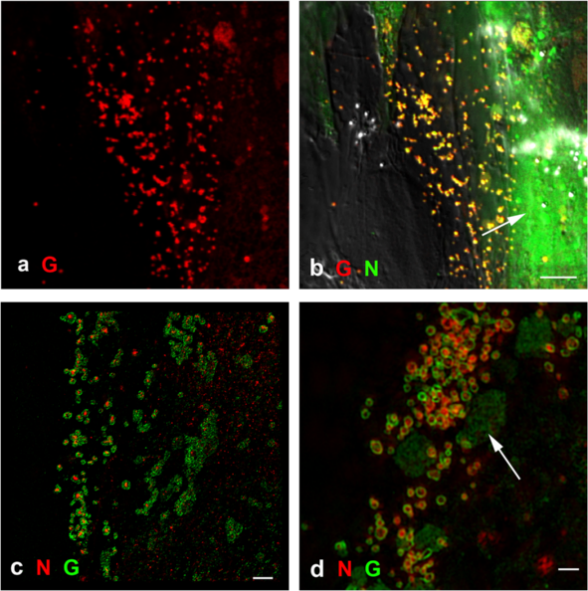
**Confocal and super-resolution microscopy: PaKi cells were infected with HeV, fixed at 18 hpi and prepared as for conventional confocal imaging. a, b)** Cells were labelled with anti- HeV G antibody detected with anti-human antibodies conjugated to Alexa 568 (**a**, red) and co-localised with rabbit anti-N antibody detected with anti-rabbit antibodies conjugated to Alexa 488 (b, green). The labelling is co-localised in dots and there is also N protein labelling within the cell cytoplasm (arrow). Cell structure is shown in image **b)** using DIC. **c)** SR GSD image of a similar culture labelled as for **a**, **b**. HeV G protein (detected with Alexa 647; (shown as green for consistency) can be seen as round circles around the viral particles and HeV N protein (Alexa 488, shown as red for consistency) is inside the particles. **d)** SIM image of cells prepared as for **a, b)**. The G protein (green) forms a ring around the particles with the N protein (red) inside. The arrow indicates an area of G protein labelling on the cell membrane. Scale bars: **a, b)** =5 μm, **c, d)** =500 nm.

Using conventional confocal microscopy small HeV G (Figure 5a, b) and HeV N positive particles (Figure 5b) were readily detected on the surface of PaKi cells. Whilst the small dots showed co-localisation of the two viral proteins, it was not possible to detect any structure within the fluorescent dots. Large areas of N labelling are seen in Figure 5b (arrow) and these correspond to the ‘lakes’ of protein seen in the HeV-infected Vero cell cytoplasm in Figure 1f.

Ground stage depletion (GSD) super-resolution microscopy permits ~100 nm lateral resolution with no improvement in z plane resolution. Figure 5c illustrates a small region of the surface of a PaKi cell showing the viral particles as round structures with G (green) on the outside and N (red) on the inside. The mean diameter of the particles was 314 nm (n = 20, SD 39).

3D-Structured Illumination Microscopy (SIM) permits a two-fold increase in resolution in all three dimensions. Figure 5d is an image of a grazing section of the cell membrane with virus-like particles clearly resolved with the G protein (green) as round circles with a mean diameter of approximately 300 nm (n = 40, SD 50). HeV N protein (red) was detected within the spherical structures confirming they were HeV viral particles. In images obtained with both systems, there were regions of HeV G protein labelling which appeared to be on the surface of the cell which may represent accumulations of either HeV G protein prior to virus assembly or virus-like particles lacking N protein about to bud from the membrane. (Figure 5d arrow).

### TEM imaging confirms round particles are typical paramyxovirus structures

**Figure 6:**
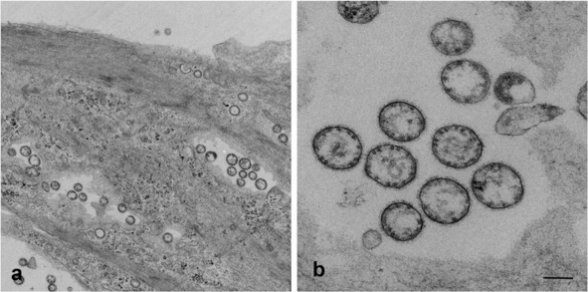
**PAEC cells infected with HeV, fixed at 18 hpi and prepared for TEM. a)** Image from section of cells taken close to the coverslip. **b)** higher magnification TEM image of virus particles showing round structure, with a viral membrane. The fuzzy surface suggests the presence of a glycoprotein layer on the outside of the virion and the RNPs are present within the virus membrane. Scale bars: **a)** =500 nm, **b)** =200 nm.

Final confirmation of the round particles seen in SR microscopy as paramyxovirus-like virions was provided by processing of HeV infected PAEC and PaKi cells for TEM imaging. Sections of infected cells showed the presence of large numbers of round particles (Figure 6a) which at higher magnification (Figure 6b) showed the typical morphology of a paramyxovirus with a fuzzy coat indicating the presence of the F and G glycoproteins on the outside of the virus, a membrane envelope and RNPs within the virus. The size of these virions was variable with a mean diameter of 284 nm (n = 20, SD 61). No pleomorphic structures similar to those detected in HeV infected Vero cells were seen.

### Is HeV M protein associated with the virus membrane or the RNP?

**Figure 7:**
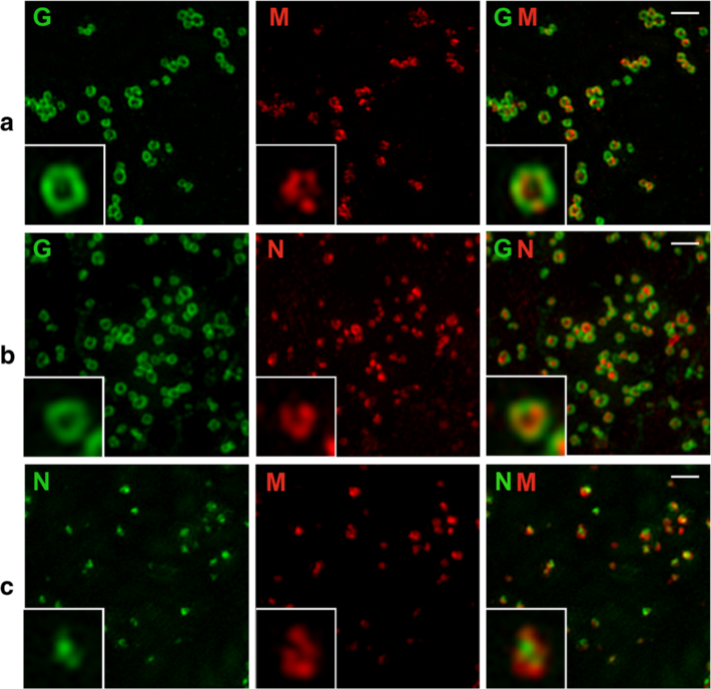
**SR SIM imaging of PAEC cells infected with HeV and fixed at 18 hpi.** Cells were labelled with anti-HeV G antibody detected with anti-human antibodies conjugated to Alexa 488, HeV M protein localised with mouse anti-M antibodies detected with anti-mouse antibodies conjugated to Alexa 568 and HeV N protein localised with rabbit anti-N antibody detected with anti-rabbit antibodies conjugated to Alexa 488 or 568. Row **a)** shows labelling of HeV G (green) with HeV M (red). Row **b)** shows labelling of HeV G (green) with HeV N (red) and row **c)** shows HeV N (green) co-labelled with HeV M (red). Insets show examples at higher magnification. Scale bars =1 μm.

To address this question, PAEC cells were infected as above and co-labelled for HeV G and M, G and N, N and M proteins and analysed by SIM SR imaging. The results indicate that the G protein consistently labels in a ring around the virions, but there were few images where M protein labelling showed a ring inside the HeV G protein (Figure 7, row a). HeV N protein formed irregular structures within the G protein ring (Figure 7 row b) and HeV M protein did appear to associate with the N protein (Figure 7 row c).

## Discussion

The process of paramyxovirus replication and virus assembly has remained ill-defined despite the importance of paramyxoviruses such as mumps and measles in human health. This has been due in part to inherent difficulties in carrying out structural analysis of pleomorphic paramyxovirus particles with proteins containing disordered regions that make them difficult to analyse. Thus, the first three dimensional structure of a paramyxovirus (Sendai virus) was not obtained until 2009 when the structure was determined using cryo-electron tomography [29]. In this structure, the nucleocapsid was free inside the virion and the matrix protein was bound to the inside of the particle. Two years later the 3-D structure of measles was obtained and showed that the matrix protein formed helices coating the ribonucleocapsid rather than the inner membrane of the virus [30]. How widespread either of these configurations is among the paramyxoviruses is not known.

Here we used a number of different techniques to help elucidate some aspects of the replication of a relatively new group of paramyxoviruses, the henipaviruses. Infection of a range of cell lines as well as primary endothelial cells gave surprisingly different results. At early stages (8 hpi) of HeV infection of Vero cells there was no sign of virus formation at the cell membrane but at 18 and 24 hpi the pattern of virus M and G protein labelling was difficult to interpret. In contrast, in A549, MDBK and HeLa cells infected with HeV and fixed at 18 hpi the majority of HeV M protein was in the nucleus with some labelling in the cytoplasm. Hendra virus G protein was present in the ER and at high levels at the cell membrane but with little co-localisation with the M protein. In these latter three cell lines, there appeared to be reduced trafficking of HeV M protein from the nucleus to the cell membrane. Whilst the defective interferon response in Vero cells [31] may complicate the interpretation of these experiments, the reasons for the apparent accumulation of the HeV M protein in the nucleus of A549, MDBK and HeLa cells remain to be resolved. Clearly, immortalised cell lines are able to produce infectious virus particles, but the patterns of HeV M and G proteins seen in these cell lines were not observed in HeV-infected endothelial cell cultures at any of the time points from 8 – 48 hrs (data not shown). What role this may play in the titres of infectious virus produced in the different cell lines remains unclear.

As endothelial cells are a major target for HeV and NiV *in vivo* [26] the results obtained with infection of these cells were seen as of particular interest in the context of the *in vivo* virus assembly process. Previous reports involving NiV have shown some differences in the infectivity of endothelial cells from different sites [32] with aortic endothelial cells refractory to infection unless modified to express the ephrin B2 receptor. This is in contrast to the results presented here where two species of primary aortic endothelial cells were readily infected with HeV. Similar results have been obtained with NiV (data not shown).

In a recent study of henipavirus replication in a number of cell lines, Aljofan et al. [27] reported that virus protein levels and viral titres varied considerably between cell types. The highest levels of virus production were obtained with Vero cells, HeLa cells being intermediate and A549 producing a lower viral titre. The authors did not address the question of the subcellular distribution of viral proteins. Our data broadly support their results and also indicate possible reasons for the differences in virus titre production they observed from different cell lines.

NiV M protein has been reported to transit the nucleus [25], but this is the first report of this occurring in HeV infected cells. The function of nuclear trafficking of henipavirus M protein is unclear but a recent report by Wang et al. [25] showed that for NiV, the M protein had both nuclear localisation and export motifs and was ubiquitinated in the nucleus. Preventing this process had dramatic negative effects on M protein trafficking within the cytoplasm and viral budding. Nuclear localisation of M protein has been reported for other paramyxoviruses including Sendai virus [23], Newcastle disease virus [24] and RSV [22]. RSV M protein, whilst present in the nucleus, is excluded from the nucleolus and its export from the nucleus relies on the crm-1 exporter receptor [21]. However the authors reported that the M protein was then found in the cytoplasm in contrast to HeV infected cells, where almost all detectable M protein appears in the form of virions at the cell membrane. The function of the process remains obscure but a role in inhibiting host cell transcription has been proposed for the M protein of RSV [21]. An unexpected observation in this study was a tendency for the cells with the highest number of virus particles to have the lowest level of HeV M protein in the nucleus. One possible explanation is that rather than a continuous synthesis and trafficking of HeV M through the nucleus during the infection process, there is a wave of M synthesis; the protein moves through the nucleus but is not continuously replaced. The mechanism and function of such a process remains highly speculative.

Many questions remain concerning the arrangement of the virus proteins within the viral particle and the structure of the virus assembly site. The arrangement of the M protein has recently been studied for measles virus and it has been suggested that it is closely associated with the RNP rather than the inner face of the viral envelope [30]. There are very few reports of the use of SR microscopy to image virus structure, although the value of SR imaging has recently been demonstrated for vaccinia virus [33]. In this study, we investigated the value of two very different SR approaches. Localization microscopy using GSDIM/dSTORM relies on the detection of single fluorophores, whose position can be determined with nanometre precision. GSDIM/dSTORM renders the majority of fluorophores transiently non-fluorescent by performing ground state depletion and reversibly transferring the molecules to an “off-state” (Triplet- and Dark-states). Due to the reversible nature of this process, nearly all fluorophores can be imaged while being transiently in their fluorescent ‘on-state’ over the acquisition time. Based on the position information of the detected fluorophores, a single super-resolution image is calculated [34,35]. In contrast, 3D-Structured Illumination Microscopy (3D-SIM) works by illuminating the sample with a known pattern (in this case a grid pattern). The grid pattern produces high frequency information in the form of moiré fringes that can be mathematically extrapolated to result in a two fold increase in resolution in x, y and z directions [36]. Despite significant differences between the two SR systems, both gave very similar images of HeV virions and demonstrated for the first time sub-particle resolution of paramyxovirus proteins and the round morphology of HeV virions. Imaging of virions co-labelled for the presence of HeV M, N and G proteins provided a first indication that the M protein appeared be associated with the RNP rather than the virion envelope.

Overall these results indicated that HeV should be described as a predominantly round virus with a diameter of 280 – 310 nm depending on imaging method. As the samples remained hydrated throughout the labelling and imaging process of SR microscopy, a virus diameter of approximately 300 nm should offer a more accurate estimate of the virus *in vivo* dimension.

Despite clear serological evidence of HeV infection, actual isolation of the virus from bats has not proved simple [37]. An additional objective of this work was to identify any differences in HeV M and G protein localisation between bat and non-bat cells. Comparison of the expression pattern of M and G proteins in HeV-infected endothelial cells with that seen in two bat-derived cell lines indicated that there was no observable difference between the two cell types in response to henipavirus infection, but a pronounced difference between these two cell types and other continuous cell lines.

## Conclusion

These findings provide novel insights into the structure of HeV. They have highlighted the benefits of SR imaging for studies of paramyxovirus structure and show that for HeV imaging studies the choice of tissue culture cells may affect the experimental results. The results obtained using several different imaging approaches indicate that HeV should be considered a predominantly round virus with a mean diameter of approximately 280 nm by TEM and 310 nm by SR imaging.

## Methods

### Preparation and culture of primary cells

Following euthanasia, approximately 3 cm pieces of aorta were collected aseptically from a 24 hr old piglet and 14 day aged calf and placed in RPMI +10% FCS. Tissues were washed in PBS + antibiotics (Pen/Strep and Fungizone) twice then treated with collagenase (Sigma Aldrich) and incubated at 37°C for 15-20 min. Cells were recovered by centrifugation at 1500 RPM for 5 min, and the pellet of cells re-suspended in 5 mL growth media (EMEM, Gibco) and added to a 25 cm tissue culture flask (Corning). The endothelial cell cultures showed an essentially homogeneous cell morphology which was maintained for up to 10 passages. All experiments were conducted on cultures of less than 6 passages. Primary cultures were immunolabelled for the presence of PECAM and were >90% positive for this cell marker (data not shown).

### Culture of cell lines

Cell lines (Vero, ATCC CCL81; A549, ATCC CCL185; HeLa, ATCC CCL2; MDBK, ATCC CCL22) were grown in EMEM with 10% calf serum (EMEM-GM) and passaged as required. They were incubated at 37°C in 5% CO_2_. The two bat-derived cell lines were prepared as previously described [28].

### Cells for confocal and super-resolution microscopy

Cells were infected at BSL-4 with a low passage of Hendra virus Redlands (Hendra virus/Australia/Horse/2008/Redlands) [38]. Cells grown in 24 well plates containing 13 mm glass coverslips were infected at an MOI of 8 and infections were stopped at the appropriate time by removing EMEM-GM and replacing it with 4% paraformaldehyde in PBS. Cells for super resolution microscopy were grown in 6 well plates containing coverslips and were infected as for confocal microscopy. Fixed cells were removed from the BSL-4 laboratory and all labelling was undertaken under normal laboratory conditions.

#### Preparation of virus inoculum for negative staining

An aliquot of a HeV inoculum prepared as described previously [39] was fixed in a 1:1 (v/v) ratio with 8% paraformaldehyde in PBS to comply with biosecurity protocols and stored at 4°C. A formvar coated grid was floated on a drop of the inoculum for 5 min and transferred to a drop of Nanodrop (Nanoprobes) for 1 min. The grid was blotted and imaged in a Philips CM120 electron microscope.

#### Processing of infected cells for TEM

Cells were seeded onto thermanox coverslips (ProSciTech) and processed for electron microscopy as previously described [40].

#### Processing of cells for confocal and SR imaging

Cells were seeded onto 13 mm glass coverslips in 24-well plates (Nunc) for confocal and SIM imaging and 18 mm square coverslips for SR GSD imaging. They were incubated overnight, and infected with HeV at an MOI of 8 as described previously. They were fixed at 18 hpi in 4% (w/v) paraformaldehyde in phosphate-buffered saline and removed from the containment area. They were stored at 4°C in PBS.

#### Fluorescence immunolabelling

Fixed cells were permeabilised with 0.1% (v/v) Triton X-100 in PBS for 10 min and washed in PBS. Non-specific labelling was blocked with a 30 min incubation in 0.5% bovine serum albumin in PBS (PBS/BSA) and all antibodies were diluted in PBS/BSA. Primary antibodies were incubated for 60 min and after 3 × 5 min washes were detected with fluorescent-conjugated species-specific immunoglobulins diluted 1:200 in PBS/BSA (Life Technologies). After 3 × 5 min PBS washes and one dH_2_0 wash, nuclei were stained with a 1:4000 dilution of DAPI (Sigma) in dH_2_0. Coverslips were mounted in Vectashield (Vector Laboratories) and sealed with nail varnish.

### Primary antibodies and fluorescent conjugates

Anti-Hendra antibodies: rabbit anti-HeV G 1:2000 (AAHL raised against recombinant NiV G protein expressed in CHO cells), human anti-HeV G (mAb m102.4 generously gifted by CC Broder) 1:1000 [41] rabbit anti-HeV N 1:2000 (AAHL, raised against recombinant NiV N protein expressed in CHO cells), mouse monoclonal anti-HeV M 1:10 (AAHL, [42]), Protein disulphide isomerase 1:1000 (Quantum Scientific), PECAM 1:50 (Santa-Cruz). Species-specific secondary antibodies were from Life Technologies and conjugated to Alexa 488, 568, 543 or 647 (1:200).

### Fluorescence imaging

For conventional confocal imaging the labelled coverslips were imaged with a Leica SP5 confocal microscope. Data collection was using Leica LAS AF. 3D-Structured Illumination Microscopy was performed on an OMX V4 3D-SIM system fitted with a 60x (1.42 NA) objective (GE Healthcare/Applied Precision). Image reconstruction was with softWoRx (GE Healthcare/Applied Precision).

For super-resolution GSDIM-imaging with the Leica SR GSD, 18 mm coverslips were stored in PBS after immunolabelling at 4°C. The coverslips were mounted onto a single depression slide (76 mm × 26 mm) and the cavity filled with approx. 100 μl PBS containing 50 mM β-Mercaptoethylamine (Sigma-Aldrich, # 30070) adjusted to pH 7.4 as imaging buffer. The imaging was performed with a Leica SR GSD system using a 100× (NA 1.47) objective and a 1.6x post magnification (160 × in total). The two fluorophores were recorded sequentially and image acquisition, single molecule analysis and image reconstruction was performed with Leica LAS AF 2.6.1.

Figures 1, 2, 3, 4, 5 and 6 were prepared using Adobe Photoshop and Figure 7 was prepared using FIJI (NIH).
